# Vitamin B12 Supplementation Adequacy in Australian Vegan Study Participants

**DOI:** 10.3390/nu14224781

**Published:** 2022-11-11

**Authors:** Amanda J. Benham, Danielle Gallegos, Katherine L. Hanna, Mary T. Hannan-Jones

**Affiliations:** 1 School of Exercise and Nutrition Sciences, Queensland University of Technology (QUT), Kelvin Grove 4059, Australia; 2 Woolworths Centre for Childhood Nutrition Research, Faculty of Health, Queensland University of Technology (QUT), South Brisbane 4101, Australia

**Keywords:** vegan, plant-based, vitamin B12, intake, absorption, supplementation

## Abstract

In our initial analysis of the Australian Vegan Study we estimated the mean daily intake of vitamin B12 of each participant and compared this to the Recommended Dietary Intake (RDI). However, the proportion of vitamin B12 that can be absorbed from large doses typically contained in oral supplements is considerably lower than the amount absorbed from food. In this analysis we took into account the estimated absorption from supplements in order to compare adequacy of vitamin B12 intake to the RDI. A cross-sectional online survey was used to obtain information from women (N = 1530) of reproductive age on a vegan diet in Australia. Vitamin B12 intake from food was estimated using a validated food frequency questionnaire and detailed questioning was used to estimate supplemental intake. We used published data on dose-dependent absorption rates to estimate amount of the vitamin absorbed to enable comparison to the RDI. Supplementation practices varied widely. Based on estimated amount absorbed, 39% of participants had an estimated total intake of vitamin B12 below RDI equivalency, compared to 26% based on mean daily intake. The potential absorption of vitamin B12 needs to be considered when estimating adequacy of intake and recommending supplementation.

## 1. Introduction

The essential nutrient vitamin B12 has traditionally been obtained from animal products by those on omnivorous and vegetarian diets. Plant-derived foods do not naturally contain a reliable source of vitamin B12 and people on a vegan diet need to consume foods with added vitamin B12 (“fortified” foods) and/or take vitamin B12 supplements. We have previously reported on the vitamin B12 sources of women of reproductive age on a vegan diet in Australia and on factors predicting intake [1]. Poor intake is a concern as deficiency can have adverse haematological and neurological consequences [2]. The effects can be irreversible or even fatal, particularly in infants, if diagnosis or treatment is delayed [3,4].

The types of foods that can allowably be fortified with vitamin B12 and the maximum amount that can be added varies between countries. In Australia, only plant milks, yeast extracts, analogues of meat and dairy products and energy drinks can have vitamin B12 added to them (at a rate of 0.2–2.0 µg per serve), at the manufacturer’s discretion [5]. The amounts that legislation allows to be added to meat and dairy alternatives is similar to the amount naturally present in the foods that these replace [5]. Based on the amount of the vitamin in commonly consumed animal products and fortified foods, meeting the RDI generally requires consumption of two to three serves per day of either fortified foods or animal products. As our initial analysis found that only 10.7% of participants met the recommended intake of vitamin B12 from fortified food alone [1], intake from supplements is of high importance in this group. Intramuscular injections of vitamin B12 are available but oral supplementation is more typical, and several forms and doses of the vitamin are available commercially. 

The estimated average requirement (EAR) of vitamin B12 is 2.0 mcg daily and the intake recommended by the World Health Organisation, the United States’ Institute of Medicine and Australia’s National Health & Medical Research Council is 2.4 mcg per day for healthy adults, increasing to 2.6 and 2.8 mcg daily during pregnancy and lactation, respectively, based on the absence of megaloblastic anemia and the presence of normal serum vitamin B12 [6,7,8]. In the United Kingdom the Reference Nutrient Intake (RNI) is 1.5 mcg daily for adults, with an additional 0.5 mcg during lactation, based on the absence of anemia [9]. However, the usefulness of these markers of adequacy to determine these recommendations used has been questioned [10,11,12]. In contrast, the European Food Safety Authority recommendations are based on normalisation of all four biochemical markers of vitamin B12 status, setting an Adequate Intake of vitamin B12 of 4 mcg per day for adults, increasing to 4.5 mcg during pregnancy and 5.0 mcg for lactating women [13]. There is no upper limit set for intake of vitamin B12 [6,7,8,14,15] and intakes of 2000 mcg per day have been used for four months to treat deficiency without adverse effects reported [16]. Analysis of National Health and Nutrition Examination Survey (NHANES) data (n = 24,262) found that high supplemental vitamin B12 intakes were not associated with higher all-cause mortality [17].

Vitamin B12 intake recommendations in Australia and the United States assume an average estimated absorption rate of 50% of the vitamin from food [6,7,8], while the European Food Safety Authority assumes 40% (11). Absorption rates from vitamin B12-fortified foods are similar to or higher than that from foods naturally containing the vitamin [18]. Absorption from food and physiological doses is predominantly via active transport of the vitamin bound to the carrier protein intrinsic factor across the brush border of the terminal ileum [19], a mechanism with capacity to transfer a maximum on average of approximately 1.5 mcg in a 4–6 h period [20,21]. Active absorption of vitamin B12 requires adequate intrinsic factor secretion as well as properly functioning ileal mucosal cells. Additionally, absorption from animal products requires properly functioning gastric parietal cells and adequate pancreatic proteolytic enzymes, as the vitamin B12 must first be cleaved from the food protein it is bound to before binding to a carrier protein [22]. Many factors can result in impaired absorption including: deficit of intrinsic factor (pernicious anemia), decreased gastric acid secretion, removal of part of the gastrointestinal tract, certain gastrointestinal diseases, parasites, high alcohol intake and use of certain medications [2,22]. The known or potential presence of any of these factors should be considered when recommendations for vitamin B12 intake are made.

Studies on subjects with pernicious anemia (who lack intrinsic factor) have found that with large oral doses of supplemental vitamin B12 there is passive absorption of the vitamin not requiring intrinsic factor [23], with approximately 0.9 to 1.2% of large doses of the vitamin (250–10,000 mcg) absorbed in both these and healthy subjects [24]. This is far less than the estimated absorption rate from food of 40–50%. Interestingly, while active absorption may be greater when vitamin B12 supplements are consumed with food (due to food stimulating the release of intrinsic factor), passive absorption has been found to be approximately 60% higher when vitamin B12 is taken on an empty stomach [24], which is considered to be due to the presence of factors in food which can inhibit absorption [18].

In our initial analysis of the Australian Vegan Study (n = 1530) participants’ mean daily intake of vitamin B12 was estimated based on reported intake from fortified foods and supplements. Comparison to recommended intakes was done without considering the variable dose-dependent absorption rates from supplements [1]. This was done in order to compare intake with that in other studies which have examined the vitamin B12 intake from supplements of vegans, where mean daily supplemental intake was added to the intake from food for assessment of intake [25,26,27]. However, the assumption that mean daily doses from supplements are equivalent to intakes from food represents a limitation which can result in a substantial over-estimation of useful vitamin B12 intake. The aims of this paper are therefore to: (i) outline the range of supplementation practices of participants in the Australian Vegan Study, (ii) compare the intake of vitamin B12 of participants to the recommended dietary intake (RDI), taking into account the estimated amount absorbed from supplements, based on the frequency and dose of supplementation and current knowledge of absorption rates and (iii) compare this to the original estimation of vitamin B12 intake based on simple mean intake and (iv) to provide limited commentary on possible appropriate supplementation regimens for those on a vegan diet.

## 2. Materials and Methods

This study was part of the Australian Vegan Study [1], which involved a survey of women of reproductive age on a vegan diet. 

### 2.1. Sample and Recruitment

Inclusion criteria for this study were: female, aged between 18 and 44 years, have been on a vegan diet (defined as a diet free of animal products) continuously for at least the past six months, intending to stay on a vegan diet indefinitely, currently resident in Australia and had done so for at least 21 months out of the past two years.

Recruitment was conducted by posting on Australian-based Facebook™ groups for people on a vegan diet. National, state-based, metropolitan and regional groups were targeted in order to recruit participants from all states of Australia, and in both metropolitan and non-metropolitan areas. After obtaining permission from group administrators a post was made inviting potential participants who met the selection criteria to complete an anonymous online survey, with a link posted to the survey information page. The survey used adaptive questioning and cookies were used to help ensure participants did not complete the survey more than once. Two prizes of vouchers valued at $250 were offered as incentives for participation. While restricting recruitment to online and via Facebook™ introduced a selection bias, 97% of Australians aged 18–44 years were internet users in 2016-17 [28] and in 2018 over 90% of Australian women aged 18–44 years were users of Facebook™ [29]. It is not possible to determine the number of potential participants that were exposed to the recruitment posts on Facebook™. However, the largest of the groups recruited from had over 60,000 members at the time recruitment took place. 

In order to determine the study size required to obtain a representative sample, an estimate was made first of the number of women aged 18–44 on a vegan diet in Australia. This was done by (i) determining the percentage of this demographic self-reporting as “vegetarian” and as “avoiding dairy products” in a 2016 consumer poll [30], and (ii) contacting the administrator of several of the largest Facebook™ groups for vegans in Australia for an estimate of the number of women on a vegan diet aged 18–44 in Australia (personal communication, S. DeSilva, 23/2/2017). The resultant estimate for the number of potential participants in late 2017 was between 70,000 and 150,000. The online calculator provided by the Australian Bureau of Statistics was utilised and using the upper end of the range as the population size, a confidence level of 95% and a confidence interval of 0.05, it was determined that the minimum sample size required for this study was 384 [31]. 

### 2.2. Data Collection

Data was collected via a cross-sectional online survey in a six-week period from April until June 2018. There were 150 questions relating to demographics, diet, health indices and behavior and intake of vitamin B12, taking approximately 20 min to complete. The Checklist for Reporting Results of Internet E-Surveys (CHERRIES) [32] was used for implementing and reporting the survey details. This paper has been prepared using the STROBE checklist for cross-sectional studies [33]. This study was conducted according to the guidelines laid down in the 1964 Declaration of Helsinki and all procedures involving human subjects were approved by the Queensland University of Technology’s Office of Research Ethics and Integrity (approval #1700000950).

Estimation of vitamin B12 intake from food and drinks was done via an adaptation of a validated food frequency questionnaire (FFQ), originally developed to estimate vitamin B12 intake of vegetarian women in New Zealand [34], where legislation regarding fortification of foods with vitamin B12 is identical to that in Australia [35]. Best practice guidelines for dietary assessment in health research were considered in adopting and adapting this tool [36], including rigorous face and content validity Questions were included on all vitamin-B12 fortified foods that were available for sale in Australia at the time of survey, identified by examination of products in supermarkets and other food stores, with assistance from five Accredited Practising Dietitians working in the field of plant-based nutrition. Products included plant-based milks and other dairy alternatives, meat analogs, yeast extracts and flakes, energy drinks and meal replacement products. These are the only foods that can be fortified with vitamin B12 under the Australian and New Zealand regulations, and the maximum level at which they can be fortified is also specified, with fortification being at the manufacturer’s discretion [35]. The vitamin B_12_ content of each product was determined from the food labels and/or contact with manufacturers. Color photographs of all 105 products identified were used in the online survey to enhance recognition. 

Participants were asked their frequency of consumption of individual vitamin B_12_-containing foods in the past three months, with nine answer options provided for frequency and five answer options for usual serve size. 

To determine vitamin B12 intake from supplements the survey included questions on the frequency of consumption and dose of vitamin B12 supplementation in the past three months, including from multi-nutrient supplements (such as multivitamins) and vitamin B12 oral supplements and injections. Questions relating to the form and type of vitamin B12 supplements taken were also included.

### 2.3. Data Preparation and Analysis

Little’s MCAR (Missing Completely at Random) test [37] and separate variance t-tests were used to investigate the pattern of missing data. Resulting *p*-values were all greater than 0.05, indicating that missing values were missing completely at random. For each estimation of total vitamin B12 intake, the percentage of total participants with missing data preventing determination of category of vitamin B12 intake was approximately 7%. Simple pairwise deletion of data was used for these analyses.

Participants’ total intake of vitamin B12 from food was determined from their FFQ answers relating to consumption of fortified foods. In the initial analysis of adequacy of total vitamin B12 intake [1], intake via supplementation was calculated without considering the variable rates of absorption of different supplemental doses, nor how these compare to absorption from food. In order to compare these initial results (based on simple mean daily intake of vitamin B12) with those obtained when taking the limited absorptive capacity of vitamin B12 from a single dose into account, the assessment of intake from oral supplements of each participant was re-evaluated. To estimate absorption rates, available evidence on the estimated amount absorbed from various crystalline doses of vitamin B12 was examined. These studies used radio-labelled vitamin B12 in different methodologies: the Schillings test, based on urinary excretion [24], the Heinle test, based on fecal excretion [38], the Glass test, based on hepatic uptake [39] and whole body radiation monitoring methods [40]. Correlation of the results between these various tests has been found to be relatively high [41].Absorption rates at various doses are shown in Table 1.

Heinrich [43] examined absorption of vitamin B12 in doses up to 10,000 mcg and from the data developed an equation (referred to herein as “Heinrich equation”) to describe total absorption from a single dose of the vitamin:

Uptake (absorption) = 1.5D/D + 1.5 + (1 − 1.5/D + 1.5) × 0.009 × D, where D = the dose.

Estimate absorption based on the Heinrich equation was closely correlated with absorption rates found by other researchers [24]. This equation was used to estimate the amount of vitamin B12 each participant absorbed from supplements. Estimated amounts absorbed derived from the equation for supplemental doses commonly used by participants are shown in Table 2.

The steps undertaken to assess vitamin B12 intake, taking into account estimated absorption rates were as shown in Table 3.

The figure calculated at Step 6 was used to categorize participants according to the estimated amount of vitamin B12 they absorbed per day on average, to determine the prevalence of meeting/not meeting recommended intakes and to compare the results to those obtained when intake of vitamin B12 was based on simple mean daily intake. As in the original analysis, for pregnant and lactating women (n = 138) the appropriate EAR, RDI and EFSA AI was used to categorize the intake of these participants.

For participants who were supplementing their vitamin B12 intake via intramuscular injections (n = 64), an estimated amount absorbed of 80% of injected dose was used, based on studies of absorption rates via this route of administration [44]. As intramuscular doses reported were all 1000 mcg, one or more injections in the past three months was considered adequate to meet the RDI. This is because even if only 20% of an intramuscular dose was absorbed/ retained, this would equate to 200 mcg/90 = 2.22 mcg per day, which is greater than the 1.2 mcg estimated amount absorbed indicated by the RDI.

Data were analysed using SPSS Version 25 (IBM Corp. 2017Armonk, North Castle, NY, USA: IBM Corp). Continuous variables were assessed for normality and the range, and as appropriate, median, mean and standard deviation were determined and reported. Categorical variables were presented as frequencies (percentages).

## 3. Results

The completed survey was submitted by 1530 participants who confirmed that they met the inclusion criteria. An additional 990 people commenced the survey but did not complete or submit it, giving a completion rate of 61%. As submission of the survey was taken as consent to participate, the results of those who did not submit the survey were not used. However, comparison of their characteristics to those that completed the survey indicated that they did not differ significantly in any way. 

Demographic characteristics and health indices and behaviours of the group have been reported previously [1].

### 3.1. Vitamin B12 Supplementation Practices of Australian Vegan Study Participants

Participants (N = 1530) reported a wide range of practices related to vitamin B12 supplementation, with 27% reporting not taking any supplements containing vitamin B12 in the past three months. Supplement use reported included the use of injected and oral supplements, of both single-nutrient and multi-nutrient supplements containing vitamin B12, different forms of cobalamin (cyanocobalamin, methylcobalamin, adenosylcobalamin and hydroxocobalamin), single doses ranging from 1 to 10,000 mcg and frequency of supplementation ranging from never to twice daily in the preceding three-month period. The reported doses of vitamin B12 in multinutrient supplements ranged from 1 to 7500 mcg, with 46% being greater than or equal to 100 mcg; the reported doses in single-nutrient B12 supplements ranged from 1 to 10,000 mcg, with 84% being 100 mcg or more. The supplementation practices of participants are summarised in Table 4.

### 3.2. Impact of Recalculations on Assessment of Adequacy of Supplemental Intake

When participants’ vitamin B12 intake from oral supplements was recalculated based on the predicted amount absorbed (and then doubled for comparison to the RDI) the estimated mean intake from supplements decreased from 261.0 to 6.5 (SD 11.7; median 2.7; range 0–203) mcg/day. Three cut-points were chosen for the purpose of classifying participants’ intake: (i) Estimated Average Requirement (EAR), which is the average daily level of intake estimated to meet the requirements of 50% of healthy individuals (ii) Recommended Dietary Intake (RDI) which is known as the Recommended Daily (or Dietary) Allowance (RDA) in the USA and is the mean daily dietary intake level that is sufficient to meet the nutrient requirements of nearly all (97–98 per cent) healthy individuals [7,8] and (iii) the EFSA Adequate Intake (AI) of 4 mcg [13], as there is evidence that this is closer to the intake at which indicators of vitamin B12 status are maintained [45,46]. The categorization of participants’ intakes based on the two methods of estimating intake from supplements is shown in Table 5.

Just over half of participants had an intake from supplements equivalent to less than the RDI based on estimated amount absorbed, compared with 32% when supplement intake was calculated as mean daily intake. Total intake did not meet RDI equivalency based on estimated absorbed for 39% of participants, compared with 26% when calculated as mean daily intake. Estimation of the percentage of those supplementing who were doing so at a level below RDI equivalency rose from 4.5% to 23.7% when based on estimated amount absorbed rather than simple mean intake. Of the participants who were pregnant (n = 44) and/or lactating (n = 101) 47.7 and 47.5%, respectively had a total intake of vitamin B12 below the RDI when supplemental absorption rate was taken into account.

## 4. Discussion

The key findings of the current study are: (i) taking into account the estimated amount of vitamin B12 absorbed from supplements results in a substantially lower estimate of “useful” intake of the vitamin compared to simply estimating mean daily intake; (ii) a quarter of participants in this study reported not taking any vitamin B12 supplements and of those supplementing, almost a quarter may not have been supplementing adequately; (iii) the recalculated estimated total intake of vitamin B12 of 39% of women on a vegan diet in Australia is below RDI equivalency and may be inadequate.

This is the first known study to explore a method for determining the adequacy of supplemental vitamin B12 intake based on the estimated amount of the vitamin absorbed. 

While vitamin B12 intake from supplements may be less important in countries such as the United States where food fortification is widespread, appropriate supplementation is especially relevant when supplemental vitamin B12 is the primary or only source of the vitamin, such as amongst vegans in countries such as Australia where fortification of foods is limited or where intake of fortified foods is low.

The finding that 39% of this group had an estimated total vitamin B12 intake below RDI equivalency when estimated absorption is taken into account is disconcerting as maintaining an adequate intake is especially important for women of reproductive age, as not only maternal deficiency but also sub-optimal intake can adversely impact infant growth and development during pregnancy and lactation [3,47]. It is especially concerning that the total vitamin B12 intake of almost half of the pregnant and lactating women was estimated as being below the RDI when absorption of supplements was taken into account, given that foetal and infant deficiency can result in permanent neurological damage.

Various forms of the vitamin in supplemental form were taken by our participants, and it is known that differences in stability, absorption and retention rates exist between different forms [48,49]. While the stability and effectiveness of oral cyanocobalamin in preventing and reversing deficiency has been established [50], the stability and effectiveness of the enzyme forms of vitamin B12, methylcobalamin and adenosylcobalamin has not been extensively studied [50,51]. A recent study reported that vegans supplementing with cyanocobalamin had significantly higher levels of the marker holotranscobalamin than those supplementing with methylcobalamin, which the authors suggested could indicate superior absorption of biologically active vitamin B12 from cyanocobalamin [52]. However, further research in this area is needed to determine the effect of various supplemental forms of the vitamin on the biomarkers of B12 activity, methylmalonic acid and homocysteine. The wide variation in supplementation practices in this group could be due to the lack of published guidelines on vitamin B12 supplementation for vegans available to both health practitioners and the public, and the wide variations between the existing guidelines. Practice guidelines and publicly available recommendations from official health authorities on supplemental intake of vitamin B12 for vegans are scant in Australia. The Nutrient Reference Values document states that vegan mothers should supplement their diets with vitamin B_12_ at the RDI level [8]. While a review of guidelines on vitamin B12 supplementation is outside the scope of this work, it appears that specific, evidence-based guidelines are also lacking in some other countries where veganism is on the rise. Neither the USA Dietary Reference Intake paper [14], the EFSA vitamin B12 paper [13] nor the UK’s National Health Service guidelines on vegan diets provide guidelines on vitamin B12 supplementation for those on vegan diets [53]. The Italian Society of Human Nutrition has proposed guidelines on supplemental vitamin B12 doses for maintenance for various age and physiological states, however the recommendations do not specify the recommended form of the vitamin, whether a stand-alone supplement is recommended and the timing of ingestion, all of which affect absorption rates [54]. A 2019 paper on vegan nutrition for mothers and children aimed at health practitioners reported the Italian Society of Human Nutrition guidelines and also included guidelines on supplemental doses to correct deficiency, depending on serum concentrations of the vitamin [55]. However, it appears that these more detailed guidelines have not been widely promulgated amongst or adopted by health practitioners. For example, the National Institutes of Health (NIH) Vitamin B12 fact sheets for health professionals [56] and consumers [57] mention the risk of vitamin B12 deficiency for vegans but do not provide any specific guidelines on supplementation.

In addition to the need for specific guidelines on dosage, there is evidence that consideration should also be given to whether supplemental vitamin B12 is in a single nutrient supplement or is in combination with other nutrients, such as in a multivitamin supplement. It has been found that other nutrients present in supplements (such as vitamin C, copper and thiamine) can render 20–90% of the vitamin B12 biologically inactive [58,59]. These combinations can also potentially form harmful analogues that can not only reduce the amount of biologically active vitamin B12 available for absorption but can potentially inhibit vitamin B12-dependent enzymes, thereby increasing the likelihood of deficiency [58,59]. A case of severe vitamin B12 deficiency in the infant of a vegan mother who had supplemented with a prenatal multivitamin containing the RDI for vitamin B12 during pregnancy raises concerns about the application of the RDIs for those who are relying wholly or partially on supplemental intake to meet their nutrient needs [60] Given what is known about the potential for vitamin B12 to become inactivated by other nutrients in supplemental form and the formation of potentially harmful analogues, it may be prudent to recommend against relying on multi-nutrient supplements as a source of vitamin B12. The finding that nearly a quarter (23%) of participants in this study were taking a multi-nutrient supplement as their only supplemental source of vitamin B12 is potentially of concern. 

Further research is needed on the absorption of various forms and formulations of vitamin B12 supplements. Based on Heinrich’s equation it appears that either a thrice daily intake of 0.5 to 1.0 mcg (in or with food) or a single daily oral dose of 5 mcg would be required to meet the equivalent to the RDI for people with no impairment to absorption. However, some absorption studies indicate that a dose of at least 20 mcg daily would be required [42]. Alternatively, a weekly dose of 1000 mcg (taken on an empty stomach) would theoretically meet the recommended intake of adults with no impediments to absorption. Higher doses may be required during pregnancy and lactation, where daily doses are more prudent due to the dependence of the foetus and infant on regular maternal intake, as infant deficiency can occur in the absence of maternal deficiency [3,47]. Much higher doses may be required to replenish diminished stores or if absorption is compromised due to disease states, medications, surgery and other factors affecting the gastrointestinal tract. Current state of knowledge suggests that best practice would be to supplement with the cyanocobalamin form of vitamin B12 in a single-nutrient supplement, taken with food if lower doses are used (to maximise active absorption) or on an empty stomach for higher doses (to maximise passive absorption). 

Various studies on vegans and non-vegans have found differing results relating to the dose of vitamin B12 required to (a) maintain satisfactory vitamin B12 status and (b) overcome deficiency. A study on vegans and vegetarians with marginal vitamin B12 deficiency found that a dose of either 50 mcg daily or 2000 mcg weekly improved metabolic markers of the vitamin to adequate levels with no significant differences between the two dosing regimens [61]. However, studies on women and infants have found that in some cases, daily supplemental doses of 50 or 250 mcg were inadequate to provide optimal vitamin B12 status during pregnancy and lactation [62,63].

While it appears clear that supplementing vitamin B12 once daily at the RDI would be inadequate to meet requirements, the results of these studies also suggest that supplementing at the level equivalent to the current RDI based on estimated amount absorbed may also be inadequate to optimise vitamin B12 status. This notion is supported by studies examining metabolic markers of the vitamin [45,46] and the higher level of intake recommended by the European Food Safety Authority (EFSA) [13]. As calculated from Table 5, 54%of the group had an estimated total vitamin B12 intake equivalent to less than the EFSA recommended intake when supplemental absorption rates were taken into account. Based on Heinrich’s equation, a daily supplemental dose of at least 25 mcg or a weekly dose of 1000 mcg would be required to meet EFSA recommendations. However, due to the lowered passive absorption when consumed with food, a recommendation of double these doses would be prudent, which corresponds to the daily recommendations of the Italian Society of Human Nutrition [57]. More research in this area would be helpful to assist in the development of specific, evidence-based guidelines on the frequency, form and dose of supplementation required to ensure vitamin B12 adequacy for people on a vegan diet.

While a strength of this study was its consideration of the vastly different rates of absorption of vitamin B12 from different supplement doses compared to food, there were limitations. The reassessment of vitamin B12 adequacy in this group did not take into account other factors known to affect absorption such as whether the supplement was in a multi-nutrient or single-nutrient formulation, whether it was taken with food or not and the form of cobalamin taken. It also did not take into account the potential effect of other factors such as race, genetic variations, reproductive status, disease states and medications known to affect vitamin B12 absorption, as insufficient is known about the quantitative effect of these. The estimation of the amount of vitamin B12 absorbed at various doses is based on the relatively limited data in this area, which was not all obtained from a similar population group. Intake of vitamin B12 from food and supplements was self-reported on a one-time basis and may not necessarily accurately reflect actual or usual intake. Although the focus of the study was on comparing reported intake to recommended intake, it would have been useful if biochemical markers were measured to determine relationships between these and reported intakes. 

## 5. Conclusions

The limited absorptive capacity for vitamin B12 in supplemental form should be considered when (i) assessing adequacy of total vitamin B12 intakes and (ii) making recommendations on supplemental intake of vitamin B12 for people on diets low in this vitamin. Not doing so could have serious consequences for the health of those on vegan diets (including infants) who rely on supplementation to meet their requirements. The extremely variable and relatively high rate of inadequate vitamin B12 supplementation found in this study could reflect an apparent lack of clear, evidence-based guidelines available to health professionals and those on vegan or other plant-based diets on assuring adequacy of vitamin B12 intake. The development and promulgation of explicit guidelines is required to help ensure adequacy of vitamin B12 intake in vulnerable groups. These could include: the type of vitamin B12 to be consumed, its consumption as a single nutrient supplement, and the dosage of the vitamin required, depending on frequency of supplementation, age and physiological state. More research is needed to clarify optimal supplementation for those on a vegan diet.

## Figures and Tables

**Table 1:** Studies on Estimation of Amount of Vitamin B12 Absorbed from a Single Oral Dose.

Single Oral Dose (mcg)	% Absorbed	Mean Estimated Amount Absorbed (mcg) ^a^	Reference
0.5	90.5	0.43	[39]
	71.0	0.36	[38]
1.0	49.2	0.49	[40]
	44.4 (MCb)	0.44	[40]
	56	0.56	[38]
2.5	50.4	1.26	[42]
5	20.4	1.02	[40]
	18.8 (MCb)	0.94	[40]
	28.0	1.4	[38]
10	16.0	1.6	[38]
20	6.0	1.2	[42]
25	5.5	1.4	[40]
	6.1 (MCb)	1.6	
50	3.0	1.5	[39]
500	2.0	10.0	[18]
	1.6 (empty stomach)	7.8	[24]
	1.0 with food	4.8	[24]
1000	1.2	12.0	[24]

^a^ cyanocobalamin form used, unless otherwise indicated. MCb = methylcobalamin.

**Table 2:** Estimated amounts of vitamin B12 absorbed for common dosages, based on Heinrich equation [24].

Dose of Vitamin B12 (mcg)	Estimated Amount Absorbed Using Heinrich Equation (Our Calculations)
1	0.6
2	0.9
5	1.2
10	1.4
20	1.6
25	1.6
50	1.9
100	2.4
200	3.3
250	3.7
500	6.0
1000	10.5
2000	19.5

**Table 3:** Steps in assessing vitamin B12 intake based on estimated amount absorbed.

Step	Calculation
1	From each participant’s stated highest dose vitamin B12 supplement, the amount absorbed from that dose was estimated, using the Heinrich equation above.
2	The amount from Step 1 was multiplied by the estimated number of times per month that the participant reported that they took this supplement, (e.g., if daily, it was multiplied by 30) and then divided by 30 to give an estimated average amount absorbed daily.
3	If the participant reported taking a second supplement containing vitamin B12, once again the dose and the “Heinrich equation” were used to estimate the average amount absorbed per day, as above.
4	The average daily amounts from Step 2 and 3 were summed to give a total estimated average amount absorbed per day.
5	To compare this amount to the EAR and RDI (which are based on 50% absorption), this amount was doubled.
6	To determine total intake to compare to the EAR and RDI, the amount from Step 5 was added to the estimated intake of vitamin B12 from food.

EAR = Estimated Average Requirement (2.4 mcg/day for lactation, 2.2 for pregnancy, 2.0 others); RDI = Recommended Dietary Intake (2.8 mcg/day for lactation, 2.6 for pregnancy, 2.4 others).

**Table 4:** Vitamin B12 Supplementation Practices of Vegan Women aged 18 to 44 years (N = 1530).

Supplementation Practices	n	% of Whole Group (N = 1530)	% of Those Supplementing (N = 1131)
Frequency of Supplementation in past 3 months:			
never	387	25.3	-
don’t know	12	0.8	-
once a month or less	72	4.7	6.4
2–3 times per month	155	10.1	13.7
1–3 times per week	402	26.3	35.6
4–6 times per week	187	12.2	16.5
every day	296	19.4	26.3
twice daily	17	1.1	1.5
Type of supplement taken:			
Takes supplement containing vitamin B12 as only nutrient	872	57.0	77.1
Takes multivitamin only	150	9.8	13.3
Takes supplement containing vitamin B12 plus other nutrient/s	110	7.2	9.7
Form of Supplement taken:			
Injections (cyanocobalamin or hydroxocobalamin)	64	4.2	5.7
Oral supplement (liquid, tablet, capsule, lozenge)	1067	69.7	94.3
Form of cobalamin (B12) taken			
Oral cyanocobalamin	516	33.7	45.6
Oral hydroxocobalamin	6	0.4	0.5
Oral adenosylcobalamin	3	0.2	0.3
Oral methylcobalamin	273	17.8	24.1
Don’t know/not answered	318	20.8	28.1

**Table 5:** Categorization of Participants According to Vitamin B12 Intake from Supplements and in Total: Comparison of Results from Different Methods.

		Intake Calculated as Mean Intake/Day [1]		Intake Recalculated as Estimated Amount Absorbed	
	from Fortified Food [1] (n = 1530)	from Supplements (n = 1419)	Total B12 Intake (n = 1426)	from Supplements (n = 1409)	from Total Intake (n = 1416)
**Intake Category**	% of group (n)	% of group (n)	% of group (n)	% of group (n)	% of group (n)
Intake = 0	2.1 (32)	27.1 (385)	0.6 (8)	27.3 (385)	0.6 (8)
>0 < EAR	81.8 (1252)	3.9 (55)	23.3 (330)	22.5 (317)	33.5 (475)
>=EAR < RDI	5.4 (82)	0.6 (8)	2.2 (31)	1.2 (17)	4.4 (63)
**Intake < RDI**	**89.3 (1366)**	**31.6 (448)**	**26.1 (370)**	**51.0 (719)**	**38.6 (546)**
>=RDI < EFSA AI	7.9 (121)	2.1 (30)	5.3 (75)	9.6 (135)	15.6 (221)
>=EFSA AI	2.8 (43)	66.4 (942)	68.6 (971)	39.4 555	45.8 (649)
**Intake Meets RDI**	**10.7 (164)**	**68.5 (972)**	**73.9 (1046)**	**49.0 (690)**	**61.4 (870)**

EAR = Estimated Average Requirement (2.4 mcg/day for lactation, 2.2 for pregnancy, 2.0 others); RDI = Recommended Dietary Intake (2.8 mcg/day for lactation, 2.6 for pregnancy, 2.4 others). EFSA AI = European Food Safety Authority Adequate Intake (5 mcg/day for lactation, 4.5 for pregnancy, 4.0 others). Note: small difference in n due to more detailed data required to categorize intake based on estimated amount absorbed.

## Data Availability

Data is held in the Queensland University of Technology data repository and is available upon written request.
